# The spectrum of thrombotic microangiopathy related to monoclonal gammopathy

**DOI:** 10.1093/ckj/sfad306

**Published:** 2024-01-08

**Authors:** Daan P C van Doorn, Myrurgia A Abdul-Hamid, Leon A M Frenken, Pieter van Paassen, Sjoerd A M E G Timmermans, for the Limburg Renal Registry

**Affiliations:** Department of Nephrology and Clinical Immunology, Maastricht University Medical Center, Maastricht, The Netherlands; Department of Biochemistry, Cardiovascular Research Institute Maastricht, Maastricht, The Netherlands; Department of Pathology, Maastricht University Medical Center, Maastricht, The Netherlands; Department of Internal Medicine, Zuyderland Medical Center, Heerlen, The Netherlands; Department of Nephrology and Clinical Immunology, Maastricht University Medical Center, Maastricht, The Netherlands; Department of Biochemistry, Cardiovascular Research Institute Maastricht, Maastricht, The Netherlands; Department of Nephrology and Clinical Immunology, Maastricht University Medical Center, Maastricht, The Netherlands; Department of Biochemistry, Cardiovascular Research Institute Maastricht, Maastricht, The Netherlands

**Keywords:** clone-directed treatment, complement dysregulation, eculizumab, monoclonal gammopathy of renal significance, thrombotic microangiopathy

## Abstract

**Background:**

Recent studies showed a high prevalence of monoclonal gammopathy (MG) in patients with thrombotic microangiopathy (TMA) aged over 50 years and suggested that complement dysregulation is pivotal for the disease to develop. Here, we studied this premise in seven patients with TMA and coexisting MG.

**Methods:**

Patients with TMA on kidney biopsy and/or peripheral blood were recruited from the prospective COMPETE cohort (NCT04745195) and Limburg Renal Registry. Patients were screened for complement dysregulation, including genetics/factor H autoantibodies (FHAA) and functional *ex vivo* testing on microvascular endothelial cells.

**Results:**

Seven (8%) out of 84 patients with TMA presented with a coexisting MG. MG clustered in patients aged over 50 years (*n/N *= 6/32, 19%). C4 and/or C3 levels were low in three patients, while four patients presented with normal complement levels. None of the patients carried rare variants in complement genes. Massive *ex vivo* C5b9 formation on the endothelium was noted in one patient; purified IgG from this patient caused massive *ex vivo* C5b9 formation via the alternative pathway of complement activation, pointing to complement dysregulation in the fluid phase. Kidney biopsies from other nephropathies linked to MG rarely exhibited concurrent TMA (*n/*N = 1/27, 4%).

**Conclusions:**

MG clustered in patients with TMA aged over 50 years. TMA and coexisting MG represents a heterogeneous disease spectrum, including a small subset of patients who may present with complement dysregulation.

## Graphical Abstract

**Graphical Abstract:**
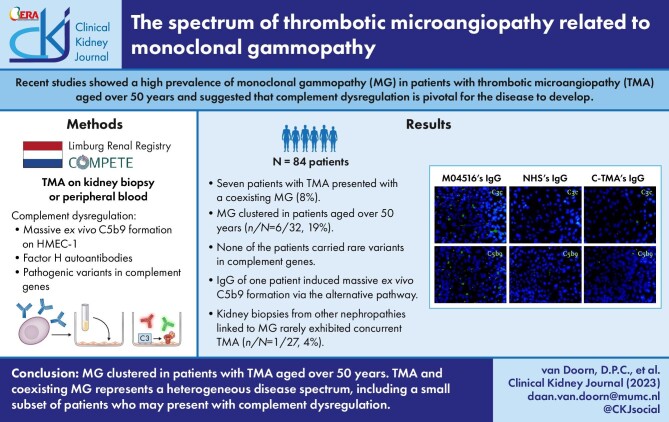

KEY LEARNING POINTS **What was known:**

- Monoclonal immunoglobulins (MIg) are prevalent in patients with thrombotic microangiopathy (TMA) aged over 50 years, suggesting a causal relation between monoclonal gammopathy (MG) and TMA.
- Preliminary data suggest that MIg may affect complement regulation, akin to complement-mediated (C-)TMA.

**This study adds:**

- Most patients with TMA and a coexisting MG present with normal complement regulation.
- Morphologic features of TMA are rarely expected in patients with other nephropathies related to MG.

**Potential impact:**

- Plasma exchange and/or therapeutic complement inhibition may not be effective for the treatment of TMA and coexisting MG.
- Future studies should focus on clone-directed treatment to control the TMA in patients with coexisting MG.

## INTRODUCTION

Thrombotic microangiopathies (TMAs) are rare, potentially life-threatening conditions that reflect tissue responses to severe endothelial damage, originating from distinct disorders, such as, complement-mediated (C-)TMA and thrombotic thrombocytopenic purpura among other etiologies [1]. TMAs typically manifest with consumptive thrombocytopenia, microangiopathic hemolytic anemia, and ischemic organ damage, often affecting the brain and kidneys. Patients with TMA should be classified according to etiology to indicate targets for treatment, having impact on treatment and prognosis.

Recent studies showed a high prevalence of monoclonal immunoglobulins (MIg) in patients with TMA aged over 50 years (i.e. ∼20% [2, 3] versus <5% in the general population [4]), suggesting a causal relation between the monoclonal gammopathy (MG) and TMA. Of note, most diseases occurring in patients with MG are considered coincidental. TMA, however, was provisionally added as an MG-associated disease by the International Kidney and MG Research Group [5]. From a hematologic point of view, many of such patients present with MG of renal significance (MGRS) and, per definition, do not present with a malignant disease requiring chemotherapeutic treatment. The mechanistic link between both entities should therefore be elucidated to indicate targets for treatment. Martins *et al*. suggested that complement dysregulation is common in patients with TMA and coexisting MG, akin to C-TMA [2]. Here, we studied this premise in seven well-defined patients with TMA and coexisting MG using routine complement measures, functional *ex vivo* endothelial cell tests, and genotyping. In addition, kidney biopsies of 27 patients with various nephropathies linked to MG were studied for morphologic features of TMA and *ex vivo* complement activation.

## MATERIALS AND METHODS

### Patient population and definitions

Patients with TMA on kidney biopsy and/or peripheral blood were recruited from the Limburg Renal Registry [6] and COMPETE cohort, a prospective observational cohort that is currently recruiting patients with TMA (NCT04745195). TMA was defined as typical morphologic features of TMA on kidney biopsy [7] and/or the triad of platelets <150 000/µL, microangiopathic hemolytic anemia (i.e. hematocrit <30%, hemoglobin <6.5 mmol/L, lactate dehydrogenase >500 U/L, and either undetectable haptoglobin or schistocytes on peripheral blood smear), and acute kidney injury. TMA was classified according to the underlying etiology [1, 8]. Patients with thrombotic thrombocytopenic purpura, defined as an enzymatic activity of von Willebrand factor cleaving protease (also known as ADAMTS13) <10% or the combination of platelets <30 000/µL and serum creatinine ≤200 µmol/L, and those with a Shiga toxin-producing *E. coli* infection were excluded.

In addition, 27 patients with various nephropathies linked to MG were randomly recruited from the Limburg Renal Registry. Patients’ kidney biopsies have been reassessed for morphologic features of TMA to study the prevalence of concurrent TMA along the spectrum of disease.

At the time of presentation before the initiation of treatment, serum samples were obtained, processed, and immediately stored at −80 degrees Celsius to prevent *in vitro* complement activation. This study has been approved by the Medical Ethics Assessment Committee (METC azM/UM; no. 2021–018) and is in accordance with the Declaration of Helsinki.

### Kidney tissue sections

Kidney tissue sections were processed for light-, immunofluorescence-, and electron microscopy, as described [6]. TMA and nephropathies linked to MG were defined according to international standards [5, 7]. The Mayo Clinic Chronicity Score (MCCS) was applied to grade chronic changes [9].

### MIg assays and hematologic definitions

MIg were detected and quantified using serum protein immunofixation (SPIF) and electrophoresis (SPEP), respectively; free light chains were quantified using a latex-enhanced immunoassay (Freelite, Binding Site, UK) [10, 11]. B-cell and plasma cell clonal disorders were classified according to international standards [12, 13]; MGRS was defined as morphologic features linked to MIg and any B-cell or plasma cell clonal disorder that did not meet criteria for a malignant disease [5].

### Routine complement measures

C4 and C3 serum levels and the functional activity of the classical and alternative pathway (Svar Life Sciences, Malmo, Sweden) were assessed.

### Rare variants in complement genes, *THBD*, and *DGKE* and factor H autoantibodies (FHAA)

Patients were screened for rare variants, i.e. variants with a minor allele frequency <0.1% according to the Genome Aggregation Database, and single-nucleotide polymorphisms in *CFH, CFI, CD46, CFB, C3, CFHR1, CFHR2, CFHR3, CFHR4, CFHR5, THBD*, and *DGKE* using DNA sequencing. Rearrangements in the *CFH*-*CFHR1*-*CFHR5* genomic region were analysed by multiplex-ligation probe amplification. The classification of variants was based on international standards [14]. Pathogenic variants, including likely pathogenic variants, were defined as those with functional studies supporting a defect in complement regulation, including null variants in genes linked to complement regulation (*CFH, CFI*, and *CD46*), variants located in a mutational hotspot, variants located in a functional domain, or variants that cluster in patients with C-TMA as demonstrated by Osborne *et al*. [15]. Benign variants, including likely benign variants, were defined as those not associated or very unlikely associated with an abnormal phenotype or increased disease risk [14]. Rare variants not fulfilling these criteria have been classified as uncertain significance.

FHAA of the IgG isotype were assessed using an enzyme-linked immunosorbent assay (VIDIA, Vestec, Czech Republic) according to the manufacturer's instructions; serum samples were tested in duplicates. The cut-off was set at 1000 AU/mL, according to the standardization committee recommendations [16].

### Functional *ex vivo* testing

*Ex vivo* C5b9 formation on resting and/or perturbed microvascular endothelial cells of dermal origin (i.e. HMEC-1; ATCC, Manassas, VA, USA) was assesses as described [17]. Briefly, HMEC-1 were plated on glass culture slides and used when >80% confluent, incubated with serum diluted in test medium for 3 hours at 37°C, fixed in 3% formaldehyde, and blocked with 2% bovine serum albumin for 1 hour. HMEC-1 were either preincubated with 10 µM adenoside diphosphate for 10 minutes to mimic a perturbed endothelium or not [18]. Rabbit anti-human C5b9 pAb (1:100; Calbiochem, San Diego, CA, USA) and Alexa488-labeled anti-rabbit Ab (1:100; Life Technologies, Carlsbad, CA, USA) were used; nuclei were stained with 4′,6-diamidino-2-phenylindole (DAPI). Fluorescent staining was acquired in 15 fields and the staining area was evaluated using ImageJ software (National Institutes of Health, Bethesda, MD, USA). The samples were compared with pooled normal human serum (NHS) run in parallel using the paired sample *t* test or Wilcoxon signed rank test (R, version 4.0.4).

To study (M)Ig-mediated complement activation on HMEC-1, IgG was purified from serum samples using Protein G Sepharose 4 Fast Flow beads (GE Healthcare, Uppsala, Sweden); elution buffer 0.1 M glycine-HCl, with a pH of 2.7, was used. IgG was dialyzed overnight using a dialysate of HBSS and tris buffer, with a pH of 7.35. Purified IgG was diluted in HBBS and 5% bovine serum albumin (1:9). The solution was diluted in pooled NHS (1:9) and incubated on perturbed HMEC-1 for 3 hours at 37°C, fixed in 3% formaldehyde, and blocked with 2% bovine serum albumin for 1 hour. FITC-labeled goat anti-human IgG pAb (1:100; ICN Biomedicals, Irvine, CA, USA), mouse anti-human C4d mAb (1:200; Quidel, Alkmaar, The Netherlands) followed by FITC-labeled anti-mouse Ab (1:60; Dako, Heverlee, Belgium), FITC-labeled C3c pAb (1:20; Dako), or rabbit anti-human C5b9 pAb (1:100; Calbiochem) followed by Alexa488-labeled anti-rabbit Ab (1:100; Life Technologies) were used; nuclei were stained with DAPI. Fluorescent staining was acquired in 15 fields and the staining area was evaluated using ImageJ software (National Institutes of Health), as is described.

### Descriptive statistics

All data were analysed using manually installed packages in R-Studio (version 4.0.4). Normally distributed variables were presented as mean ± standard deviation (SD). Skewed variables were presented as medians (interquartile range; IQR). Categorical variables were presented as counts and percentages.

## RESULTS

### Characteristics of patients with TMA and coexisting MG

The serum of 84 out of 113 patients with TMA was available for screening for MG. Seven (8%) out of 84 patients presented with coexisting MG, either MGRS (*n *= 6) or multiple myeloma (MM, *n *= 1); hypercalcemia, lytic bone lesions, and κ free light chains >13.7 g/L (ratio, >2015) defined MM. ADAMTS13’s enzymatic activity was >10% in six patients tested for; the other patient presented with platelets of 245 × 10^9^/L, excluding thrombotic thrombocytopenic purpura [19]. MG, indeed, clustered in patients with TMA aged over 50 years (*n*/*N *= 6/32, 19%). Patients’ characteristics and complement tests have been depicted in Table 1. Severe acute kidney injury was noted in all patients; two (29%) out of seven patients presented with coexisting hypertensive emergency characterized by myxoid intimal edema on kidney biopsy, either with papilledema or not. Morphologic features of acute TMA [i.e. thrombosis, microaneurysms, mesangiolysis, endothelial swelling/denudation, subendothelial flocculent material, and/or (myxoid) intimal swelling] and/or chronic TMA (i.e. double contours of capillaries, onion skinning, and/or occlusive intimal fibrosis) were found on kidney biopsy. The median MCCS was 4 (IQR, 2–6). MGRS lesions other than TMA were not found. One patient (M02722) presented with extrarenal manifestations of TMA (i.e. stroke and purpura).

**Table 1:** Patients with thrombotic microangiopathy and coexisting monoclonal gammopathy.

Patients	B011	M04516	M09719	M00920	M02722	COM004	COM017
Age, years	55	44	70	66	74	74	71
Sex, M/F	M	F	F	M	F	F	F
Blood pressure, mmHg	Unknown	220/120	155/67	241/173	154/81	128/70	138/52
SCr at presentation, µmol/L	371	645	115	575	145	907	172
eGFR, mL/min/1.73 m^2^	16	7	44	9	31	4	25
Dialysis	–	+	+	–	–	–	+
MAHA	+	–	–	–	+	+	+
Platelets, ×10^9^/L	<150	339	201	245	278	35	63
TMA on kidney biopsy	ND	+	+	+	+	ND	ND
Extrarenal manifestations	–	–	–	–	+	–	–
SPIF	IgGλ	IgGκ	IgGκ	IgGλ	IgMκ	κ FLC	IgGκ, IgMλ
SPEP, g/L	<2	<2	<2	3.5	<2	>13.7	<2
Hematologic disorder	MGRS	MGRS	MGRS	MGRS	MGRS	MM	MGRS
ADAMTS13’s enzymatic activity, %	>10	76	62	ND	73	64	18
Complement measures							
Low C4	–	–	+	–	+	–	+
Low C3	–	–	+	–	–	–	–
Massive *ex vivo* C5b9 formation on resting/perturbed endothelium	–/–	+/+	–/–	–/–	–/–	–/–	–/–
Genetic variant(s)	–	–	*C3* *^a^* c.4,855A > C	*DGKE* *^a^* c.654C > T	–	ND	–
Minor allele frequency, %	N/a	N/a	>0.1	<0.01	N/a	N/a	N/a
Loss of CFHR1 and CFHR3	–	–	Heterozygous	Homozygous	–	ND	–
FHAA, AU/mL	–	1171 ± 126	2050 ± 74	–	–	–	–
Follow-up, months	5	51	31	22	16	22	1
Treatment	None	PEX, Ecu	None	None	MMF, GC	PEX, Dara-VMP	PEX, Ecu
SCr at last visit, µmol/L	Unknown	796	110	690	98	93	Dialysis
Kidney outcome	CKD G4	ESKD	CKD G3b	CKD G5	CKD G3a	CKD G3a	ESKD
Survival	Died	Alive	Alive	Alive	Alive	Alive	Died

a Variants were classified as benign.

CKD: chronic kidney disease; Dara-VMP: daratumumab, bortezomib, melphalan, and prednisolone; DGKE: diacylglycerol kinase epsilon; Ecu: eculizumab; ESKD: end-stage kidney disease; F: female; FHAA: factor H autoantibodies; FLC: free light chains; GC: glucocorticoids; MAHA: microangiopathic hemolytic anemia; M: male; MGRS: monoclonal gammopathy of renal significance; MM: multiple myeloma; MMF: mycophenolate mofetil; N/a: not applicable; ND: not determined; PEX: plasma exchange; SCr: serum creatinine; SPEP: serum protein electrophoresis; SPIF: serum protein immunofixation.

Three patients were treated with plasma exchange followed by eculizumab (*n *= 2) or clone-directed treatment, that is, daratumumab, bortezomib, melphalan, and prednisolone (*n *= 1). One patient was treated with mycophenolate mofetil and prednisolone. The other patients received conservative treatment. The median follow-up period was 22 (IQR, 5–31) months. Patients invariably progressed to chronic kidney disease, including two patients with end-stage kidney disease (ESKD). None of the patients with MGRS progressed to a hematologic malignancy. Two patients died from their underlying condition, either pancreatic cancer or disseminated colorectal cancer.

The clinical characteristics, treatment, and outcomes of patients with TMA and coexisting MG extracted from previous publications have been depicted in Table S1 (see online supplementary material).

### Characteristics of patients with nephropathies related to MG

Light chain cast nephropathy was associated with concurrent arteriolar TMA on kidney biopsy in one patient with MM; serum-induced *ex vivo* C5b9 formation on perturbed endothelium indicated normal complement regulation. The patient (M03612), a 67-year-old woman presented with platelets of 76 G/L and acute kidney injury (i.e. serum creatinine 2090 µmol/L), but no hemolysis. Dialysis was initiated and kidney function did not recover despite clone-directed treatment, that is, bortezomib and dexamethasone. Two months after presentation, the patient died on the background of severe pneumonia.

None of the patients with AL amyloidosis (*n *= 7), cryoglobulinemic glomerulonephritis (*n *= 6), light chain deposition disease (*n *= 4), proliferative glomerulonephritis with monoclonal immune deposits (*n *= 1), light chain proximal tubulopathy (*n *= 1), or ‘monoclonal’ fibrillary glomerulopathy (*n *= 1) presented with coexisting TMA. The patients’ clinical and morphologic features on kidney biopsy have been depicted in Table 2. Patients presented with coexisting MM (*n *= 14), smoldering MM (*n *= 3), MGRS (*n *= 7), chronic lymphatic leukemia (*n *= 2), or lymphoproliferative disorder (*n *= 1). The median MCCS of the control cohort was 2 (IQR, 0–3), with most patients having minimal or mild chronicity. No between group differences were found.

**Table 2:** Control patients with nephropathies associated with a monoclonal gammopathy

Patient no.	Age/sex	Diagnosis on kidney biopsy	TMA on LM	TMA on EM	MCCS	Hematologic disease	SPIF	SPEP, g/L	SCr, µmol/L	SAlb, g/L	uProt, g/24h	Massive *ex vivo* C5b9 formation on perturbed endothelium
M03221	59/F	AL	No	ND	0	MM	IgGκ; κFLC	18.8; <0.1	72	30.5	5.0	No
M05821	78/F	AL	No	No	5	MGRS	λFLC	0.1	304	24.7	8.7	No
M12421	79/F	AL	No	No	1	CLL		<0.1	70	25.1	5.0	No
M01822	76/M	AL	No	No	3	MGRS	IgGκ	2.4	376	26.8	6.7	No
M05622	72/M	AL	No	No	1	MM	IgGκ	1.1	163	15.5	4.1	No
M07822	71/M	AL	No	No	7	MM	IgGκ; κFLC	13.0; 2.2	243	41.5	0.4	No
M12222	85/M	AL	No	No	1	MM	IgAλ; λFLC	<2; <0.1	81	10.6	3.2	No
M03612	67/F	CN	Yes	ND	2	MM	λFLC	3.2	2090	29.4	3.3	No
M08516	74/M	CN	No	ND	0	MM	λFLC	1.0	1225	43.0	2.2	No
M02212	60/M	CN	No	No	3	MM	κFLC	16.2	419	44.5	5.4	No
M06712	56/F	CN	No	No	4	MM	κFLC	12.3	518	47	4.2	No
M07512	63/F	CN	No	No	2	MM	κFLC	5.2	451	39.2	4.1	No
M00713	60/M	CN	No	No	4	MM	λFLC	11.5	163	43.8	11.0	No
M09213	56/M	CN	No	No	0	MM	λFLC	10.2	733	33.8	9.7	No
M12118	69/M	CryoGN, I	No	No	2	LPD	IgMκ; κFLC	12.1; 0.6	335	34.3	5.2	No
M02020	65/F	CryoGN, I; CLL infiltration	No	No	0	CLL	IgGκ	<2	107	23.3	4.4	No
M07214	81/F	CryoGN, II	No	No	0	MGRS	IgMκ	N/a	282	N/a	1.2	No
M06913	58/M	CryoGN, II	No	ND	2	MGRS	IgMκ	0.5	179	29.4	7.7	No
M08314	60/F	CryoGN, II	No	No	0	MGRS	IgMκ	0.5	211	18.2	3.5	No
M08315	81/F	CryoGN, II	No	No	0	MGRS	IgMκ; κFLC	1.0; 0.7	143	ND	1.4	No
M09407	72/F	FGN	No	ND	3	SMM	IgGλ; λFLC	1.1; <0.1	217	22.3	4.1	No
M07217	75/F	LCPT	No	No	4	MM	IgGλ; λFLC	<2; 4.5	332	41.1	11.7	No
M13319	42/F	MIDD/LCDD	No	No	2	MM	κFLC	1.6	457	36.8	1.7	No
M07614	60/M	MIDD/LCDD	No	No	5	MM	IgAκ; κFLC	<2; 2.2	307	32.6	0.1	No
M08817	73/M	MIDD/LCDD	No	No	3	SMM	IgMκ; κFLC	<2; 1.1	170	38.9	1.8	No
M09119	72/F	MIDD/LCDD	No	No	2	SMM	κFLC	0.5	274	ND	0.1	No
M07811	59/F	PGNMID	No	No	3	MGRS	IgGκ	<2	369	25.8	4.8	No

AL: light chain amyloidosis; CLL: chronic lymphatic leukemia; CN: cast nephropathy; CryoGN: cryoglobulinemic glomerulonephritis; EM: electron microscopy; F: female; FGN: fibrillary glomerulonephritis; FLC: free light chains; LCDD: light chain deposition disease; LCPT: light chain proximal tubulopathy; LM: light microscopy; LPD: lymphoproliferative disorder; MCCS: Mayo Clinic Chronicity Score; M: male; MGRS: monoclonal gammopathy of renal significance; MIDD: monoclonal immune deposition disease; MM: multiple myeloma; ND: not determined; NHS: normal human serum; SAlb: serum albumin; SCr: serum creatinine; SMM: smoldering multiple myeloma; SPEP: serum protein electrophoresis; SPIF: serum protein immunofixation; uProt: urinary proteinuria.

### Complement measures

Four (57%) out of seven patients with TMA and coexisting MG presented with normal levels of C4 and C3, 2 (29%) patients had low levels of C4 alone, and one (14%) patient had low levels of C4 and C3. *Ex vivo* C5b9 formation on the resting endothelium showed normal complement activation in six (86%) out of seven patients; similar results were found when serum samples were incubated on the perturbed endothelium. M04516 presented with massive *ex vivo* C5b9 formation on the resting endothelium, indicating unrestrained complement activation akin to C-TMA [8, 17]. M04516’s serum, however, showed FHAA. Of note, M09719 also presented with FHAA. None of the patients carried rare variants in complement genes.

**Figure 1:**
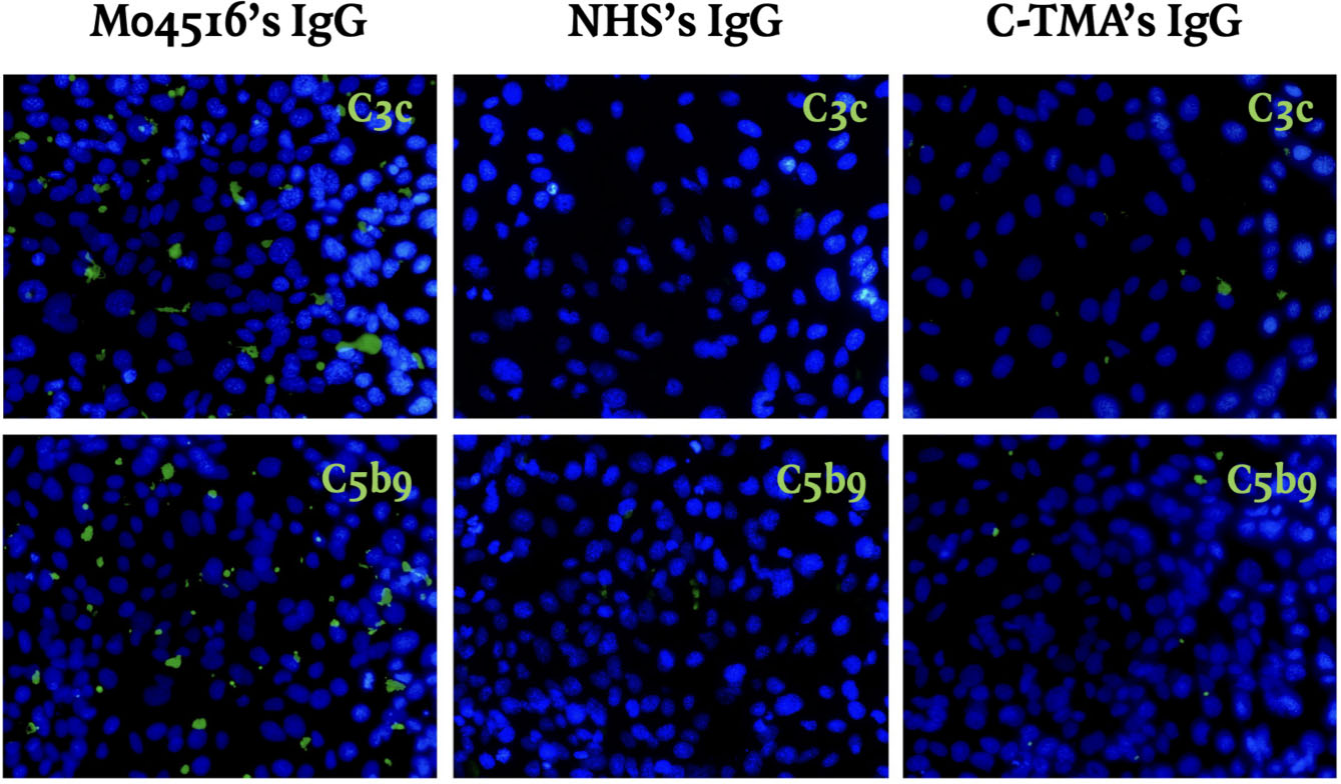
M04516’s IgG induced massive *ex vivo* complement activation via the alternative pathway (green) on the perturbed endothelium as compared to purified IgG from NHS; original magnification, ×400. Incubation of purified IgG from a patient with ‘hereditary’ C-TMA did not induce massive *ex vivo* complement formation on perturbed endothelium, similar to purified IgG from NHS. Nuclei were stained with 4′,6-diamidino-2-phenylindole (blue).

To test whether MIg and/or FHAA affect complement regulation, IgG was purified from four patients with TMA and coexisting MG (i.e. M04516, M09719, M00920, and COM004), one patient with ‘hereditary’ C-TMA on the background of a pathogenic variant in *CFI* (i.e. c.355G > A; p.Gly119Arg) and variant of uncertain significance in *CFI* (i.e. c.1397C > T; p.Thr466Ile), and pooled NHS. M04516’s IgG induced massive *ex vivo* C3c (241% as compared to NHS; *P* < 0.001) and C5b9 (182% as compared to NHS; *P* < 0.001) formation on the perturbed endothelium (Fig. 1). Neither IgG nor C4d, a marker of complement activation via the classical and/or lectin pathway, were found on the endothelium, suggesting that FHAA bind to factor H in the fluid phase. Purified IgG from the other patients, including M09719, did not affect complement activation (Table 3).

**Table 3:** *Ex vivo* depositions of IgG, C4d, C3c, and C5b9 on perturbed HMEC-1 after incubation of IgG from patients with thrombotic microangiopathy (TMA) and coexisting monoclonal gammopathy (*n *= 4) and ‘hereditary’ C-TMA (*n *= 1) as compared to healthy controls

	B11	M04516	M09719	M00920	M02722	COM004	COM017	C-TMA
IgG	ND	NS	NS	NS	ND	NS	ND	NS
C4d	ND	NS	ND	ND	ND	ND	ND	ND
C3c	ND	241%***	NS	NS	ND	NS	ND	NS
C5b9	ND	181%***	NS	NS	ND	NS	ND	NS

*** *P *< 0.001 versus healthy controls.

NS, not significant.

ND, not done.

The 27 control patients with nephropathies related to MG presented with normal *ex vivo* C5b9 formation on the perturbed endothelium (Table 2), indicating normal complement regulation. Therefore, none of these patients were screened for rare variants in complement genes and/or FHAA.

## DISCUSSION

This study describes the clinical characteristics and complement measures, including genetics/FHAA and functional *ex vivo* testing, of seven patients with TMA and coexisting MG. Patients invariably presented with severe acute kidney injury, either with systemic hemolysis or not, and hence, a kidney biopsy was needed to detect the TMA in four (57%) cases. The TMA in such patients is an acute and non-relapsing disease. All but one patient presented with coexisting MGRS. TMA was associated with normal complement regulation in most patients, as is illustrated by normal *ex vivo* C5b9 formation on the perturbed endothelium. None of the patients presented with rare variants in complement genes. TMA is rather uncommon in patients with MG and may be related to specific features of that particular MIg.

MG clusters in patients with TMA aged over 50 years, with a prevalence of ∼20% as compared to <5% in the age-matched population, corroborating previous observations [2, 3]. Most patients with TMA and coexisting MG, that is ∼75%, present with MGRS [2, 20]. ESKD appeared common (i.e. ≥45%) and the optimal treatment regimen remains to be established. Knowledge of MG's mechanism that leads to TMA is therefore warranted. Of note, our treatment-naive patient with TMA and coexisting MM achieved a (partial) renal response upon clone-directed treatment, suggesting that the MIg is causally linked to the TMA, as has been demonstrated in patients with C3 glomerulopathy and coexisting MG [21]. Martins *et al*. showed that patients may present with complement dysregulation, either related to rare variants in complement genes and/or FHAA (*n*/*N *= 9/24, 38%) [2]. Moreover, purified IgG from some of their patients (*n/N *= 4/7, 57%), including a single patient with a variant of uncertain significance in *CFI*, induced massive *ex vivo* complement activation on the endothelium. Our observations, however, are at odds with those of Martins *et al*. [2].

In our case series, serum from a single patient caused massive *ex vivo* complement activation on the endothelium on the background of a factor in the patient's IgG fraction. No activation markers of the classical and/or lectin pathway were found on the endothelium, indicating that M04516’s IgG fraction affects regulation of the alternative pathway. M04516 presented with severe acute kidney injury (i.e. serum creatinine >500 µmol/L) and moderate interstitial fibrosis/tubular atrophy on kidney biopsy, which might explain the patient's dire prognosis despite the initiation of eculizumab. Therefore, it remains to be studied whether or not complement activation is causally linked to the dire prognosis of TMA and coexisting MG. Previous studies showed no kidney response in ∼70% of patients with TMA and coexisting MG treated with therapeutic complement inhibition, with progression to ESKD in half of the patients [2, 3]. The efficacy of therapeutic complement inhibition among these patients is therefore lower as should be expected in ‘true’ C-TMA [8, 22, 23], pointing to normal complement regulation in most patients. The significance of FHAA, although found in a subset of patients [2], remains controversial because most patients lack the homozygous deletion of *CFHR1* [24], and present with low levels of FHAA. Moreover, M09719’s serum and isolated IgG did not affect *ex vivo* complement activation despite the presence of high levels of FHAA. Our observations suggest that TMA, in most patients, arises from a (direct) effect of the MIg on the endothelium and/or impaired regulation of thrombosis, whereas complement dysregulation may be involved in a small subset of patients.

The rationale for the use of plasma exchange is the removal of the MIg, assuming that MIg play a critical role in the disease. In line with previous observations [2], however, plasma exchange appeared not sufficient for the treatment of TMA and coexisting MG. Few cases suggest that clone-directed treatment, often directed against plasmacytic cells, is effective [20, 25], although publication bias should be kept in mind. COM004, who presented with MM, achieved a very good partial (hematologic) response that was followed by an improvement in kidney function. The potential benefit of clone-directed treatment should be balanced against the risks, particularly in frail patients [2]. None of the six patients with MGRS progressed to a malignant B- or plasma-cell disorder.

In conclusion, TMA and coexisting MG represents a heterogeneous disease spectrum, including a small subset of patients who present with complement dysregulation. The precise role and causal relationship of the MG in the development of TMA should be studied to better guide treatment decisions.

## Supplementary Material

sfad306_Supplemental_File

## Data Availability

Descriptive data can be provided upon request.
