# Supplementary material for: The spectrum of thrombotic microangiopathy related to monoclonal gammopathy
Source: Clin Kidney J. 2024 Jan 8;17(1):sfad306. doi: 10.1093/ckj/sfad306 (PMC10797488; doi:10.1093/ckj/sfad306)
Supplement: sfad306_Supplemental_File [file sfad306_supplemental_file.docx]

**Supplementary Materials**

*The Spectrum of Thrombotic Microangiopathy Related to Monoclonal Gammopathy*

Daan P.C. van Doorn,^1, 2, *^ Myrurgia A. Abdul-Hamid,^3, *^ Leon A.M. Frenken,^4^ Pieter van Paassen,^1, 2, #^ and Sjoerd A.M.E.G. Timmermans,^1, 2, #^; for the Limburg Renal Registry.

^1^Dept. Nephrology and Clinical Immunology, Maastricht University Medical Center, Maastricht, The Netherlands.

^2^Dept. Biochemistry, Cardiovascular Research Institute Maastricht, Maastricht, The Netherlands.

^3^Dept. Pathology, Maastricht University Medical Center, Maastricht, The Netherlands.

^4^Dept. Internal Medicine, Zuyderland Medical Center, Heerlen, The Netherlands.

^*^ Share first authorship

^#^ Share last authorship

Correspondence: Daan P.C. van Doorn, MD

Dept. Nephrology and Clinical Immunology, Maastricht University Medical Center

P. Debyelaan 25, 6229 HX Maastricht, The Netherlands

E-mail: [daan.vandoorn@maastrichtuniversity.nl](mailto:daan.vandoorn@maastrichtuniversity.nl)

For submission to *Clinical Kidney Journal*, original research article.

**Table S1.** Review of the literature on thrombotic microangiopathy and coexisting monoclonal gammopathy.

|  | **Ali *et al.* 2016.^1^** | **Vos *et al.* 2016.^2^** | **Mahmood *et al.* 2017.^3^** | **Chugh *et al.* 2017.^4^** | **Ravindran *et al.* 2017.^5^** | **Yui *et al.* 2019.^6^** | **Filippone *et al.* 2021.^7^** | **Martins *et al.* 2022.^8^** |
| --- | --- | --- | --- | --- | --- | --- | --- | --- |
| Patient(s), count | 1 | 3 | 1 | 1 | 20 | 9 | 3 | 24 |
| Age, years | 54 | N/a | 62 | 42 | 63 (range, 22 – 80) | 66 (range, 44 – 79) | 69 (range, 54 – 84) | 64 (range 30 – 85) |
| Sex, M/F | M | N/a | F | M | 13/7 | 6/3 | 3/0 | 14/10 |
| SCr at presentation, µmol/L | 269 | N/a | 274 | 274 | 356 (range, 62 – 1,238) | 292 (range, 133 – 601) | 248 (range, 80 – 522) | 363 (range, 124 –1,707) |
| Dialysis, *n*/*N* (%) | Yes | 0/3 (0) | No | Yes | 11/20 (55) | ND | 1/3 (33) | 17/24 (71%) |
| Platelets, ×10^9^/L | 81 | N/a | 63 | 74 | 128 (range, 11 –369) | 147 (range, 49 –282) | 209 (range, 178 –248) | 110 (range, 28 – 277) |
| TMA on kidney biopsy, *n*/*N* | Yes | 3/3 | Yes | Yes | 15/15 | 8/8 | 3/3 | 22/22 |
| Coexisting lesions | C4 DDD | – | No | No | – | – | – | – |
| Extrarenal manifestations, *n*/*N* (%) | Yes | 0/3 (0) | No | No | N/a | N/a | 0/3 (0) | 16/24 (63) |
| SPIF | IgGκ | IgM | IgAλ | IgGκ | IgG>IgM>IgA, κ>λ | IgG>IgM | IgG>IgM, κ | IgG>IgM>IgA, λ>κ |
| SPEP, g/L | 6.0 | N/a | 10.0 | 22.0 | 11.4 (range, 5 – 25) | 16.6 (range, 5 – 49) | ND | 5.5 (range, 2 – 25) |
| Hematologic disorder | MGRS | WM (*n*=3) | MGRS | MM | MGRS (*n*=15), MM (*n*=1), other (*n*=4) | MGRS (*n*=3), MM (*n*=5), other (*n*=1) | MGRS (*n*=3) | MGRS (*n*=18), MM (*n*=2), other (n=6) |
| ADAMTS13’s enzymatic activity, % | 49 | ND | 99 | 57 | <10 (*n/N*=2/11) | <10 (*n/N*=0/1) | <10 (*n/N*=0/2) | <10 (*n/N*=0/?) |
| Complement measures |  |  |  |  |  |  |  |  |
| Low C4, *n*/*N* (%) | No | N/a | No | No | 5/14 (36) | 1/5 (20) | 0/3 (0) | 2/24 (8) |
| Low C3, *n*/*N* (%) | No | N/a | Yes | No | 4/14 (29) | 0/6 (0) | 1/3 (33) | 7/24 (29) |
| Genetic variant(s), *n*/*N* (%) | ND | ND | *CFH* c.1825 G>A^*^ | ND | ND | 0/2 (0) | 0/3 (0) | 3^**^/17 (18) |
| Minor allele frequency, % | N/a | N/a | <0.01 | N/a | N/a | N/a | N/a | <0.01 (*n/N*=2/3) |
| FHAA, *n*/*N* (%) | ND | ND | No | ND | ND | ND | ND | 6/22 (27) |
| Follow-up, months | 14 | N/a | 24 | 1 | 35.6 (range, 0.3 –302.4) | ND | 6.5 (range, 1.0 –13.5) | 17 (range, 0.5 – 216) |
| Treatment | PEX | N/a | PEX, CyBorD | PEX | Variable | Variable | Clone-directed treatment | Variable |
| SCr at last visit, µmol/L | ESKD | N/a | 124 | ESKD | 133 (range, 71 –248) | ND | 195 (range, 124 –283) | ND |
| Kidney outcome(s) | ESKD | N/a | Partial renal remission | ESKD | Complete renal remission, *n/N*=3/20 (15%); partial renal remission, *n*/*N*=1/20 (5%); ESKD, *n/N=* 9/20 (45%); no response to eculizumab  (*n/N* =1/1) | Resolution of TMA without recurrence, *n*/*N*=7/9 (78%); death, *n/N*=1/9 (11%) | Complete renal remission, *n/N*=0/3 (0%), partial renal remission, *n/N*=1/3 (33%). | Complete renal remission, *n*/*N*=2/24 (8%); partial renal remission, *n*/*N*=6/24 (25%); ESKD, *n/N=*14/24 (58%); no response to eculizumab (*n/N*=5/8) |
| Survival | Alive | N/a | Alive | Died | Alive, *n/N*=20/20 (100%) | Alive, *n/N*=6/9 (67%) | Alive, *n/N*=3/3 (100%) | Alive, *n/N*=17/24  (71%) |

^*^Variant of unknown significance. ^**^Pathogenic variants (*n*=2) and variant of uncertain significance (*n*=1).

CKD, chronic kidney disease. CyBorD, cyclophosphamide, bortezomib, dexamethasone. C4 DDD, C4 dense deposit disease. Ecu, eculizumab. ESKD, end-stage kidney disease. F, female. FHAA, factor H autoantibodies. M, male. MGRS, monoclonal gammopathy of renal significance. MM, multiple myeloma. N/a, not applicable. ND, not determined. PEX, plasma exchange. SPEP, serum protein electrophoresis. SPIF, serum protein immunofixation. TMA, thrombotic microangiopathy. WM, Waldenström macroglobulinemia.

**Table S2.** Sera from patients with nephropathies related to monoclonal gammopathies did not induce massive *ex vivo* C5b9 formation on the perturbed endothelium, suggesting normal complement regulation.

| **Patient no.** | ***Ex vivo* C5b9 formation on perturbed endothelium, % vs. pooled NHS** |
| --- | --- |
| M03221 | 108% |
| M05821 | 163% |
| M12421 | 78% |
| M01822 | 79% |
| M05622 | 148% |
| M07822 | 86% |
| M12222 | 102% |
| M03612 | 79% |
| M07811 | 58% |
| M08516 | 90% |
| M02212 | 92% |
| M06712 | 69% |
| M07512 | 119% |
| M00713 | 86% |
| M09213 | 98% |
| M12118 | 46% |
| M02020 | 78% |
| M07214 | 74% |
| M06913 | 13% |
| M08314 | 52% |
| M08315 | 59% |
| M13319 | 108% |
| M07614 | 86% |
| M08817 | 70% |
| M09119 | 111% |
| M09407 | 74% |
| M07217 | 10% |

NHS, normal human serum

**Supplemental References**

1. Ali A, Schlanger L, Nasr SH, Sethi S, Gorbatkin SM. Proliferative C4 Dense Deposit Disease, Acute Thrombotic Microangiopathy, a Monoclonal Gammopathy, and Acute Kidney Failure. *Am J Kidney Dis*. Mar 2016;67(3):479-82. doi:10.1053/j.ajkd.2015.10.020

2. Vos JM, Gustine J, Rennke HG, et al. Renal disease related to Waldenström macroglobulinaemia: incidence, pathology and clinical outcomes. *Br J Haematol*. Nov 2016;175(4):623-630. doi:10.1111/bjh.14279

3. Mahmood U, Isbel N, Mollee P, Mallett A, Govindarajulu S, Francis R. Monoclonal gammopathy of renal significance triggering atypical haemolytic uraemic syndrome. *Nephrology (Carlton)*. Feb 2017;22 Suppl 1:15-17. doi:10.1111/nep.12934

4. Chugh S, Kichloo A, Jafri F, Yusvirazi L, Lerner R. Multiple Myeloma as the Underlying Cause of Thrombotic Microangiopathy Leading to Acute Kidney Injury: Revisiting a Very Rare Entity. *J Investig Med High Impact Case Rep*. Jul-Sep 2017;5(3):2324709617732797. doi:10.1177/2324709617732797

5. Ravindran A, Go RS, Fervenza FC, Sethi S. Thrombotic microangiopathy associated with monoclonal gammopathy. *Kidney Int*. Mar 2017;91(3):691-698. doi:10.1016/j.kint.2016.09.045

6. Yui JC, Garceau D, Jhaveri KD, et al. Monoclonal gammopathy-associated thrombotic microangiopathy. *Am J Hematol*. Oct 2019;94(10):E250-e253. doi:10.1002/ajh.25569

7. Filippone EJ, Newman ED, Li L, Gulati R, Farber JL. Thrombotic Microangiopathy, an Unusual Form of Monoclonal Gammopathy of Renal Significance: Report of 3 Cases and Literature Review. *Front Immunol*. 2021;12:780107. doi:10.3389/fimmu.2021.780107

8. Martins M, Bridoux F, Goujon JM, et al. Complement Activation and Thrombotic Microangiopathy Associated With Monoclonal Gammopathy: A National French Case Series. *Am J Kidney Dis*. Feb 22 2022;doi:10.1053/j.ajkd.2021.12.014
